# Defining safety parameters for first-trimester malaria treatment trials: stakeholder views from Real-Time Delphi survey

**DOI:** 10.1186/s12936-026-06045-1

**Published:** 2026-07-18

**Authors:** Sandra Bivegete, Hellen Barsosio, Myriam El Gaaloul, Kassoum Kayentao, Eve Worrall, Stephan Duparc, Wiweka Kaszubska, Innocent Valea, Jenny Hill, Feiko ter Kuile, Stephanie Dellicour

**Affiliations:** 1 Department of Clinical Sciences, Liverpool School of Tropical Medicine (LSTM), Pembroke Place, Liverpool, L3 5QA UK; 2 Centre for Global Health Research (CGHR), Kenya Medical Research Institute (KEMRI), P.O. Box 1578, Kisumu, 40100 Kenya; 3 MMV Medicines for Malaria Venture, Route de Pré-Bois 20, 1215 Geneva, Switzerland; 4 University of Sciences, Techniques and Technologies of Bamako (USTTB), Bamako, Mali; 5 Institut de Recherche en Sciences de la Santé/Unité de Recherche Clinique de Nanoro (CNRST/IRSS), Ouagadougou, Burkina Faso; 6 Department of Clinical Sciences, Liverpool School of Tropical Medicine, Pembroke Place, Liverpool, UK

## Abstract

**Background:**

In 2022, the World Health Organisation recommended artemether-lumefantrine as the first-line treatment for uncomplicated malaria in first-trimester pregnancy. However, emerging drug resistance and WHO’s recommendation for treatment diversification necessitate research on alternative artemisinin-based combination therapies (ACTs). We conducted a Real-Time (RT) Delphi survey to establish stakeholder views on safety endpoints and non-inferiority margins for a randomised controlled trial evaluating ACTs in first-trimester pregnancy.

**Methods:**

An international RT Delphi study was conducted between September and October 2024, including clinical researchers, regulators, policymakers, ethicists, and healthcare practitioners. Stakeholders completed an online survey, rating the importance of composite endpoints and maternal and infant safety-related outcomes on a 5-point Likert scale. Outcomes were ranked, and acceptable non-inferiority safety margin were explored. Quantitative responses were summarised descriptively, and free-text comments were analysed thematically.

**Results:**

Of the 54 stakeholders invited, 30 responded (56%). Most (23/30, 77%) agreed that a composite safety endpoint consisting of miscarriage, stillbirth, and major congenital anomalies was informative for evaluating ACT safety in the first trimester. Maternal outcomes rated as critical included maternal death (27/30, 90%), severe maternal anaemia (23/30, 77%), renal failure (20/30, 67%), and maternal intensive care admission (19/29, 66%). For infant outcomes, major congenital anomalies (28/30, 94%), stillbirth (26/30, 87%), and neonatal death (25/30, 83%) were considered essential. Most respondents endorsed a non-inferiority margin of HR = 1.2 as a pragmatic balance between trial feasibility and acceptable uncertainty around safety. Qualitative analysis highlighted support for the composite endpoint to address competing risks. However, concerns were raised regarding the non-specificity of some outcomes, such as preterm birth and small-for-gestational-age, which may be influenced by factors unrelated to treatment.

**Conclusions:**

This study provides a framework for researchers to determine key safety outcomes and acceptable non-inferiority margins for malaria treatment trials in the first trimester. Incorporating stakeholder perspectives into trial design can support evidence-informed policy and facilitate the deployment of a wider range of antimalarial treatments considered safe in early pregnancy.

**Supplementary Information:**

The online version contains supplementary material available at https://doi.org/10.1186/s12936-026-06045-1.

## Introduction

Malaria in pregnancy is a significant public health concern, with over 120 million pregnancies at risk annually in malaria-endemic areas globally [1]. In 2023 alone, an estimated 12 million pregnant women in sub-Saharan Africa were infected with malaria [2]. It is estimated that more than half of these infections occur in the first trimester, a critical period for embryonic and fetal development [3]. Malaria infections during this early stage of pregnancy can cause serious complications, including pregnancy loss, impaired fetal growth, pre-eclampsia, and severe maternal anaemia [4–12].

Until recently, the treatment of uncomplicated malaria in the first trimester was hindered by a lack of sufficient safety data on artemisinin-based combination therapies (ACTs) [13]. This limitation led to reliance on quinine, which is poorly tolerated and requires a complex 7-day regimen with thrice-daily dosing; together, these factors result in low adherence and suboptimal efficacy. In November 2022, the World Health Organisation (WHO) revised its guidelines, recommending artemether-lumefantrine (AL) as the first-line treatment for uncomplicated falciparum malaria in the first trimester, based on evidence from an individual patient data meta-analysis of observational studies spanning over two decades [14, 15]. Nevertheless, emerging resistance to ACT partner drugs, including lumefantrine, has been documented in East Africa, threatening the long-term effectiveness of AL [16]. To mitigate resistance, WHO now recommends treatment diversification, meaning more women of child-bearing potential and pregnant women may receive alternative ACTs with less established safety profiles in early pregnancy [17].

There is, therefore, a pressing need to generate high-quality evidence on the safety and efficacy of alternative ACTs in the first trimester of pregnancy. Some ACTs, such as dihydroartemisinin-piperaquine (DP) and pyronaridine-artesunate (PA), offer potential advantages over AL, including extended post-treatment prophylaxis and simpler dosing schedules, potentially improving treatment adherence. Although a treatment trial comparing DP, artesunate-mefloquine, and AL was conducted in Thailand, including all trimesters of pregnancy and 46 first-trimester participants, no such randomised controlled trials have yet been conducted in Africa [18]. Key barriers to conducting such trials include the lack of agreement on which safety endpoints are most critical to measure, what level of risk is acceptable compared to the current standard of care and how to balance potential benefits against unknown risks. The ethical implications of conducting RCTs in early pregnancy require careful consideration and broad stakeholder agreement on these parameters.

To address these challenges, we conducted an international Real-Time (RT) Delphi study to elicit key stakeholders’ opinions on:

1. Essential safety endpoints for trials evaluating antimalarial treatments in first-trimester pregnancy;
2. Acceptable non-inferiority margins for a composite safety endpoint.

This study was designed to inform the development of SAFIRE (Safety of Antimalarials in FIRst trimestEr), the first multicentre adaptive platform trial to evaluate ACTs in first-trimester pregnancy in Africa (PACTR202505891635396).

## Methods

### Study design

We conducted a web-based RT Delphi survey using the Surveylet platform (Calibrum Inc., Utah, USA) between September 9 and October 4, 2024. RT Delphi was selected over the traditional multi-round approach because it enables real-time display of aggregated responses, continuous iteration without predefined rounds, reduced participant burden, and anonymous sharing of perspectives among geographically dispersed stakeholders.

The study was designed and reported following the CREDES (Conducting and REporting DElphi Studies) guidelines [19].

### Panel selection

We identified potential stakeholders through our professional networks in malaria research and maternal health, members of relevant professional organisations and committees, and authors of key publications in relevant fields.

Inclusion criteria required relevant expertise and minimum experience thresholds for each stakeholder group (Table 1). Exclusion criteria included individuals who did not provide consent or had language barriers. Potential conflicts of interest were documented but did not constitute automatic exclusion. Purposive sampling was used to ensure representation across five stakeholder groups (Table 1).

**Table 1:** RT Delphi stakeholder group and inclusion criteria

Stakeholder group	Inclusion criteria
Clinical researchers	• Must have a minimum of five years of experience in clinical research with demonstrated expertise in designing, conducting, or analysing clinical trials. (add as a separate bullet) Prior involvement in clinical trials or observational studies on antimalarial treatments or pregnancy-related.
Policymakers	• Currently working or having worked within the last two years in health policy development at national or international levels • Experience in maternal health programming or malaria programming
Ethicists	• Must have a professional background in bioethics, medical ethics, or related fields • Should have experience with ethical issues in clinical trials or maternal health
Healthcare practitioners	• Active practice in regions with high malaria incidence • Minimum of three years of experience in maternal or child health
Pharmaceutical industry representative	• Professionals with expertise in regulatory practices, pharmacovigilance, or drug development • Should have experience with the development of treatments used in low-income countries (e.g., antimalarials, antiretrovirals)

We aimed to recruit approximately 40 stakeholders (range 25–50) across the stakeholder groups (5–10 per group). Potential participants were reviewed for appropriate expertise and ranked within each category. The top 10 stakeholders in each category were invited to participate; if they did not respond after two contact attempts, subsequent stakeholders from the ranked list were invited in their place.

### Invitation and consent process

Each identified stakeholder received a personalised email invitation containing a brief study rationale and overview, estimated time commitment, a unique link to the online survey platform, and contact information for the research team.

Upon clicking the survey link, participants were directed to an online informed consent form. Participants could only access the survey after actively indicating their consent. The platform automatically recorded the consent date and time. Those who declined consent or closed the browser were thanked for their time and exited the survey.

### Data collection

The survey instrument was uploaded into the Surveylet platform, and the research team conducted pilot testing to assess automation, user flow, and ease of navigation [20].

The survey consisted of three sections: first, a questionnaire collecting demographic and professional background information; second, Delphi questions on trial safety endpoints; and third, Delphi questions on the acceptability of safety non-inferiority margins for the composite safety endpoint. The survey questionnaire is available in Supplemental File 1.

For Sections 2 and 3, participants rated items on a 5-point Likert scale:

1. Strongly Disagree (or Not at all important)
2. Disagree (or Slightly important)
3. Neither Agree nor Disagree (or Moderately important)
4. Agree (or Very important)
5. Strongly Agree (or Extremely important)

Free-text comments were encouraged throughout to capture participants’ reasoning. Participants were informed in the introductory email and participant information sheet that after submitting their initial ratings, they would have the opportunity to view aggregated responses from the panel, including summary statistics and anonymised comments. This allowed participants to reflect on the collective feedback and adjust their responses if they wished. Reminders were sent to the panel every two weeks to ensure active engagement, prompting participants to review the shared feedback and contribute further. The survey remained open for four weeks.

### Analysis

Descriptive statistics (frequencies, proportions) were calculated for each safety endpoint and non-inferiority margin. Responses were categorised using the 5-point Likert scale described above. To investigate opinion diversity, responses were stratified by geographic context (malaria-endemic versus non-endemic countries) and stakeholder group (clinical researchers versus others). The “others” group comprised healthcare providers, policymakers, regulators, ethicists, and a pharmaceutical representative. As this RT-Delphi study sought to capture a broad range of opinions across the stakeholder groups, we did not apply a formal consensus threshold. However, a scoring of ≥ 4 (i.e. very important or extremely important) was considered to indicate high agreement.

Safety outcomes ranking was analysed by calculating mean scores and 95% confidence intervals for each endpoint, allowing direct comparison of perceived importance.

Free-text responses were analysed using qualitative content analysis. Two researchers (SB, SD) independently reviewed responses and identified key themes, which were then organised by professional background.

### Ethics

The study was approved by the Liverpool School of Tropical Medicine Research Ethics Committee (protocol #24-034) and conducted in accordance with the Declaration of Helsinki. All participants provided informed consent electronically before accessing the survey.

## Results

Of the 54 invited stakeholders, 30 participated in the RT Delphi survey, yielding a 56% response rate. Seven explicitly opted out of the survey, while 17 did not respond (Figure S1).

### Participant characteristics

Table 2 shows the participants’ professional and demographic profiles. The respondent panel consisted mainly of clinical researchers (20/30, 67%), healthcare practitioners (3/30, 10%), regulators (2/30, 7%), policymakers (2/30, 7%), ethicists (2/30, 7%), and a pharmaceutical industry representative (1/30, 3%). Gender distribution was relatively balanced, with slightly more men (17/30, 57%) than women (11/30, 37%). The majority of participants (19/30, 63%) were aged 55 years or above and held advanced degrees, with most having professional or doctoral qualifications (27/30, 90%). The geographical distribution of participants showed international representation across multiple regions, with over half (20/30, 67%) from malaria-endemic countries (Figure S2).

**Table 2:** Demographics and professional background of RT Delphi participants

Characteristic	Overall		Clinical researchers^‡^		Other^†^	
	N	%	n	%	n	%
All stakeholders	30	100	20	67	10	33
**Gender**						
Female	11	37	5	25	6	60
Male	17	57	13	65	4	40
Prefer not to say	2	7	2	10		
**Age**						
≤ 54 years	10	33	5	25	5	50
≥ 55 years	19	63	14	70	5	50
Prefer not to say	1	3	1	5		
**Education**						
Bachelor’s degree/Master’s	2	7			2	18
PhD/Professional^*^	27	90	19	100	8	73
Prefer not to say	1	3			1	9
**Regions of participants’ work location**						
Africa	17	57	10	53	7	64
Asia & Oceania	7	23	7	37	0	0
Europe & North America	6	20	2	11	< 5	36
**Endemicity of participants’ location**						
^** §**^Malaria-Endemic	20	67	13	68	7	64
^** ¶**^Non-Malaria Endemic Countries	10	33	6	32	< 5	36

^*^One healthcare practitioner also identified as a clinical researcher

^†^Other: policymakers, regulators, healthcare practitioners, ethicists, and a pharmaceutical industry representative. Data were collapsed due to small sample sizes and risk of participant identification

^‡^E.g., MD, PharmD

^§^Malaria-endemic countries: Burkina Faso, Congo, Gambia, Kenya, Malawi, Mali, Mozambique, South Africa, Tanzania, Thailand

^¶^Non-malaria-endemic countries: Australia, Belgium, France, Switzerland, United Kingdom, United States of America

### Composite safety endpoint

Two composite safety endpoints were presented as possible outcomes for assessing the safety of antimalarials in first-trimester pregnancy. The first composite endpoint (Composite 1) included miscarriage, stillbirth, and major congenital anomalies. The second composite endpoint (Composite 2) was broader and included miscarriage, stillbirth, major congenital anomalies, preterm birth (PTB), small-for-gestational-age (SGA), low birth weight (LBW), and neonatal death.

**Fig. 1:**
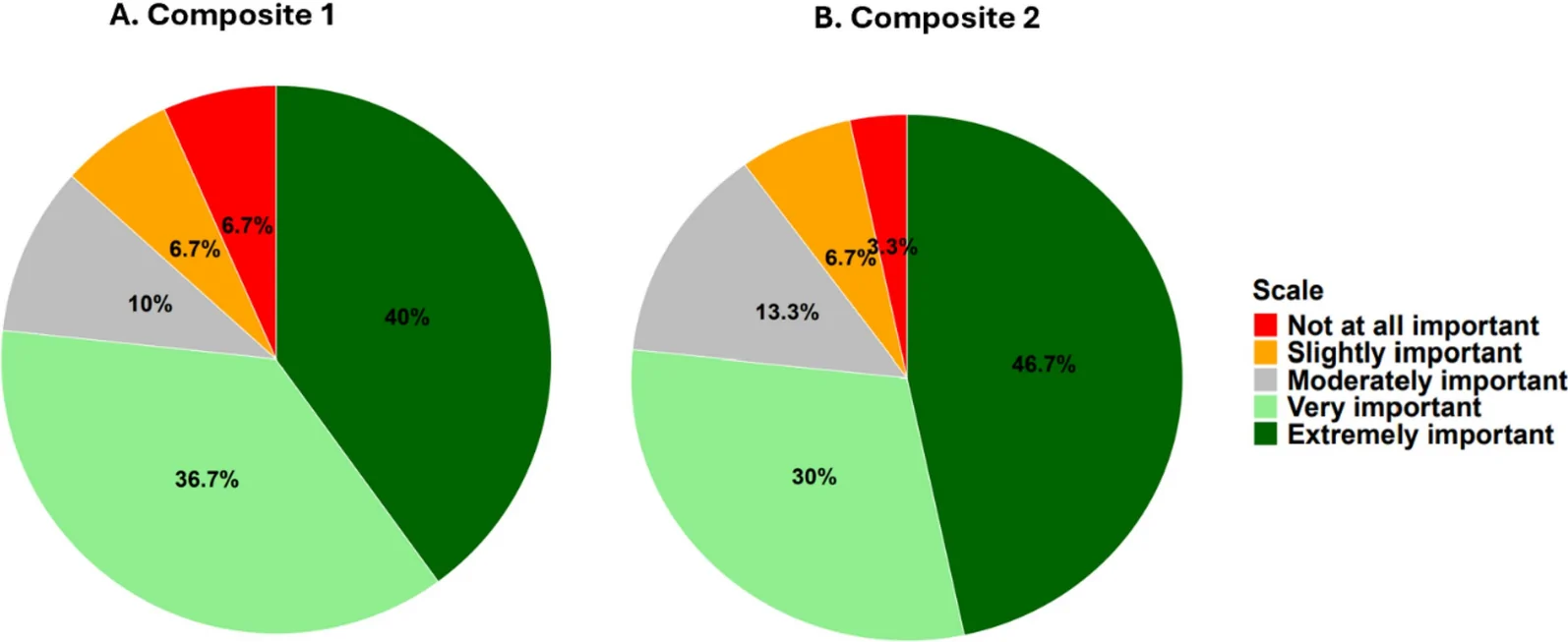
Survey responses for two composite safety endpoints for the safety assessment of antimalarial in the first trimester in treatment trials. **A** Perceived importance of Composite 1 (miscarriage, stillbirth, and major congenital anomalies) among survey participants. **B** Perceived importance of Composite 2 (miscarriage, stillbirth, major congenital anomalies, preterm birth, small-for-gestational-age, low birth weight, and neonatal death) among survey participants. Responses were measured on a 5-point scale from “Not at all important” (1) to “Extremely important” (5). Results are displayed as proportional percentages (N = 30)

Stakeholders expressed strong support for Composite 1 with 67% (20/30) strongly agreeing and 20% (6/30) agreeing that it is an informative outcome for first-trimester malaria treatment trials, while 10% (3/30) neither agreed nor disagreed, and 3% (1/30) disagreed. In a subsequent question, the majority of stakeholders (23/30, 77%) regarded it as extremely or very important, as shown in Fig. 1A. Responses were similar across malaria-endemic and non-endemic respondents (Figure S3). Agreement rates were similar among clinical researchers (15/19, 79%) and other stakeholders (8/11, 73%; Figure S4).

**Fig. 2:**
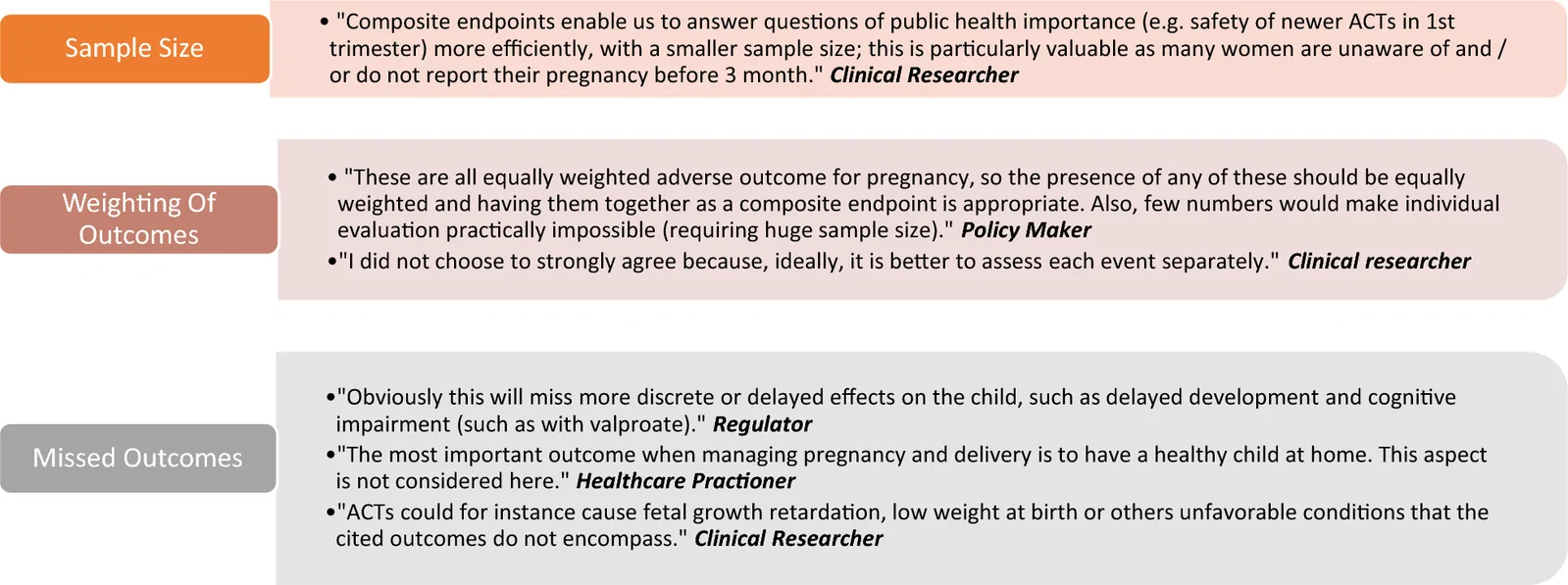
Key themes identified from participants’ responses on the use of a composite safety endpoint, including miscarriage, stillbirth, and major congenital anomalies, to assess the safety of antimalarial treatment in the first trimester

Clinical researchers valued Composite 1 for its ability to address statistical power limitations associated with relatively rare outcomes (Fig. 2). In addition, other stakeholders, including healthcare practitioners, policymakers and regulators, emphasised the importance of assigning equal weight to outcomes such as stillbirth, miscarriage, and major congenital anomalies due to their similar clinical severity. They also noted that composite endpoints are helpful for handling competing risks, as major congenital anomalies incompatible with life may lead to pregnancy loss and are thus captured by this composite. Conversely, some clinical researchers expressed concerns about equal weighting, highlighting that outcomes such as stillbirth and major congenital anomalies carry greater clinical severity compared to miscarriage and therefore should not be weighed equally. Additionally, clinical researchers, healthcare practitioners, and policymakers and regulators cautioned that using this composite safety endpoint should not overlook other critical safety outcomes not included in it, such as fetal growth restriction, SGA, PTB, or neurodevelopmental delays.

Stakeholders also expressed strong support for Composite 2 (Fig. 1B). Of the 30 stakeholders who responded, 47% (14/30) rated Composite 2 as extremely important, 30% (9/30) as very important, and 23% (7/30) as moderately to not at all important. There was no major difference between non-malaria-endemic (8/10, 80%) and malaria-endemic stakeholders (15/20, 75%; Figure S5). Clinical researchers (16/19, 84%) showed greater support than other stakeholders (7/11, 64%; Figure S6).

**Fig. 3:**
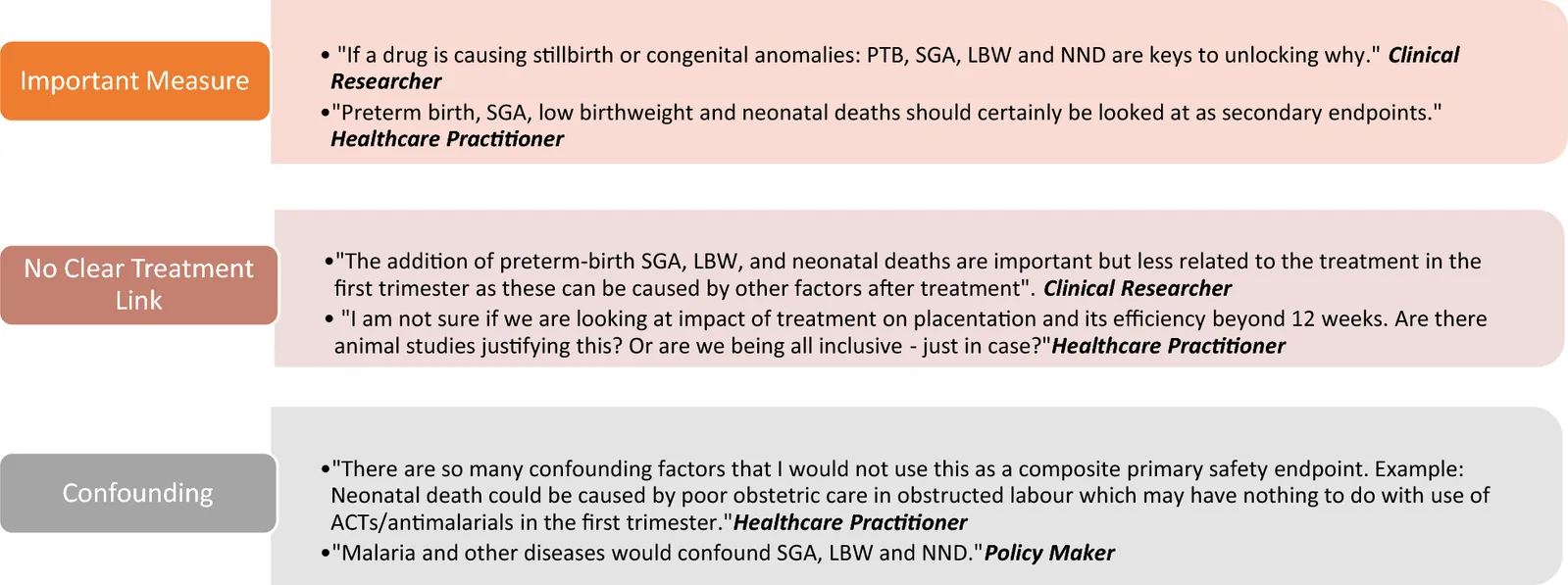
Key themes identified from participants’ rationales on the use of Composite 2, including miscarriage, stillbirth, major congenital anomalies, preterm birth, small-for-gestational-age, low birth weight, and neonatal death, to assess the safety of antimalarial treatment in the first trimester

Some stakeholders considered Composite 2 a useful metric because it includes a comprehensive set of safety-related outcomes. For example, a clinical researcher commented that evaluating PTB, SGA, LBW, and neonatal death could provide insight into the pathophysiology of adverse outcomes like miscarriage and congenital anomalies (Fig. 3). Conversely, some healthcare practitioners and clinical researchers questioned the utility of Composite 2, suggesting that the included outcomes are often influenced by multiple factors unrelated to treatment. Echoing this sentiment, a policymaker and healthcare practitioner noted that the outcomes in Composite 2 lack specificity for drug safety assessment, making it challenging to establish a direct causal link with first-trimester antimalarial exposure.

### Individual safety endpoints

#### Maternal safety-related outcomes

**Fig. 4:**
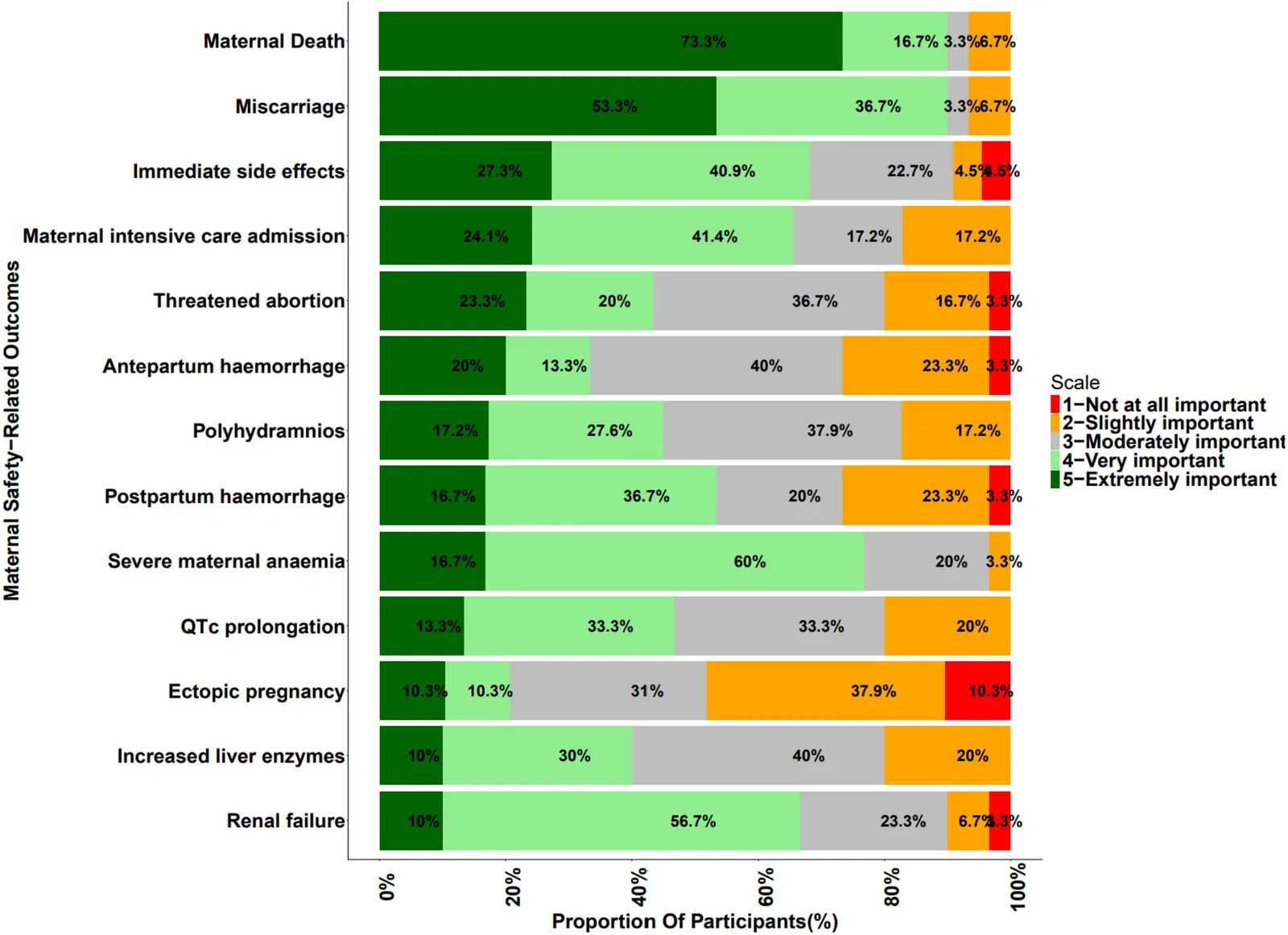
Stakeholder opinions on the relevance of maternal outcomes to assess the safety of antimalarials used in the treatment of malaria in the first trimester of pregnancy in clinical trials

Stakeholders’ perceptions of maternal safety outcomes varied considerably, with maternal death (27/30, 90%), severe maternal anaemia (23/30, 77%), immediate side effects (15/22, 68%), renal failure (20/30, 67%), maternal intensive care admission (19/29, 66%), and postpartum haemorrhage (16/30, 53%) deemed most important for assessing antimalarial treatment safety (Fig. 4). Regional analysis showed distinct differences between stakeholders from malaria-endemic and non-endemic regions, particularly regarding ectopic pregnancy, antepartum haemorrhage, postpartum haemorrhage, and threatened abortion, which were considered lower priority among stakeholders from non-malaria-endemic regions (Figure S7).

Stratification of results by stakeholder group revealed that other (i.e. non-"clinical researchers") consistently assigned higher importance to maternal outcomes than clinical researchers (Figure S8). Clinical researchers identified miscarriage, severe maternal anaemia, maternal death, renal failure, and maternal intensive care admission as crucial outcomes (Figure S8). In contrast, they assigned lower importance to ectopic pregnancy (7/18, 39%), postpartum haemorrhage (6/19, 32%), antepartum haemorrhage (5/19, 26%), increased liver enzymes (4/19, 21%), and QTc prolongation (4/19, 21%).

**Fig. 5:**
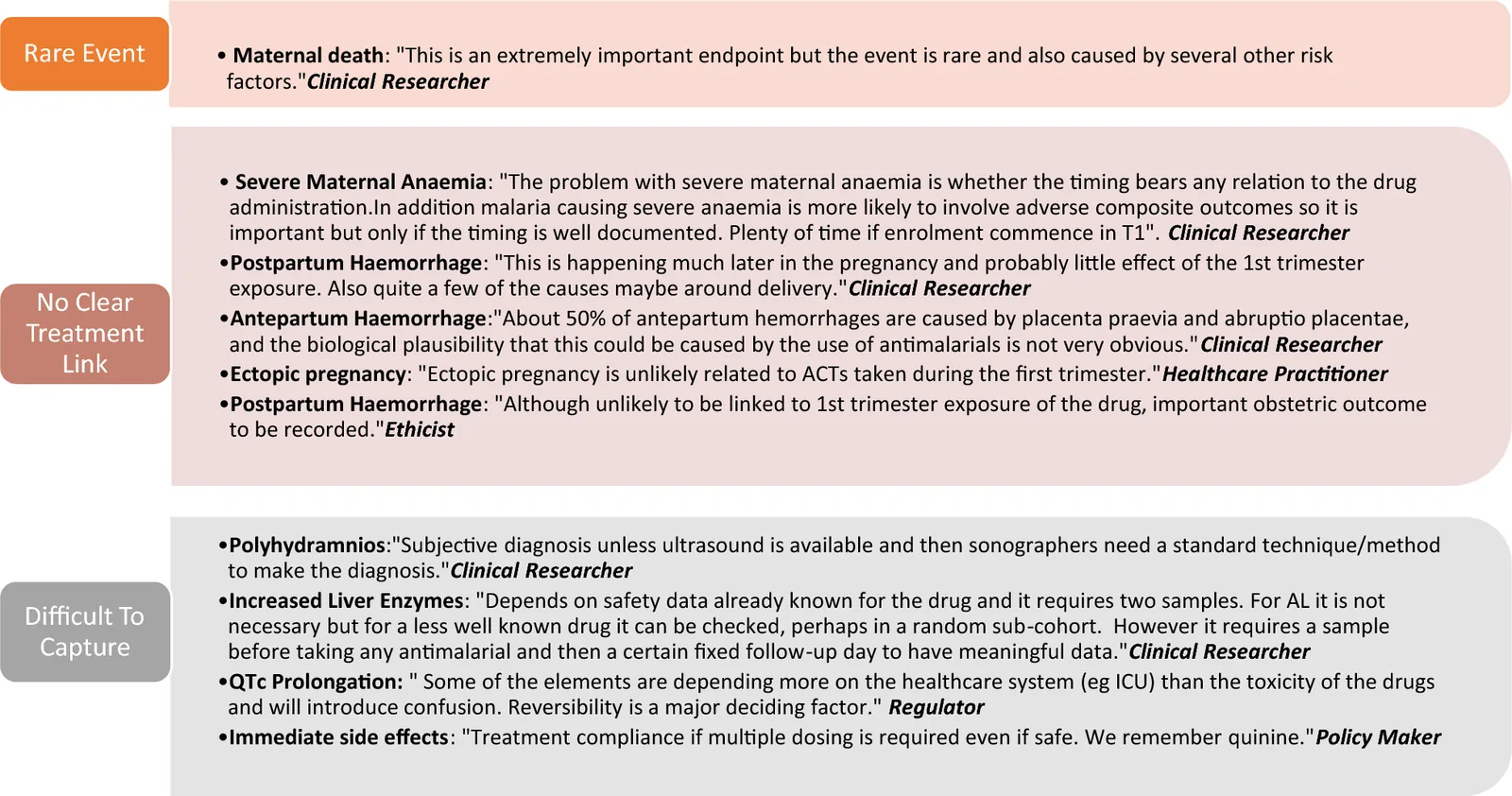
Common themes in stakeholder views on maternal-related safety outcomes in the context of first-trimester malaria treatment trials

Stakeholders held diverse perspectives on specific maternal outcomes, as illustrated in Fig. 5. For instance, maternal death was considered rare and therefore a difficult outcome to assess for evaluating new antimalarial treatments, whereas severe maternal anaemia was largely regarded as an important safety outcome. A clinical researcher noted the need to consider the timing of outcomes relative to antimalarial exposure in the first trimester. Several researchers expressed concern that outcomes such as antepartum and postpartum haemorrhage, which typically occur later in pregnancy, may be less relevant to assessing first-trimester treatment effects. Additionally, some outcomes (e.g., polyhydramnios and QTc prolongation) were deemed challenging to capture accurately due to diagnostic difficulties in resource-limited settings. It was suggested that immediate side effects may adversely affect treatment adherence and completion, thus reducing the exposure of the fetus to the consequences of partially treated malaria. Some stakeholders suggested that careful evaluation of treatment timing and drug safety profile is necessary when assessing outcomes like maternal intensive care admission, increased liver enzymes, and renal failure (Fig. 5). Ectopic pregnancy was not viewed as relevant, as it is not typically linked to antimalarial treatment.

#### Infant safety-related outcomes

**Fig. 6:**
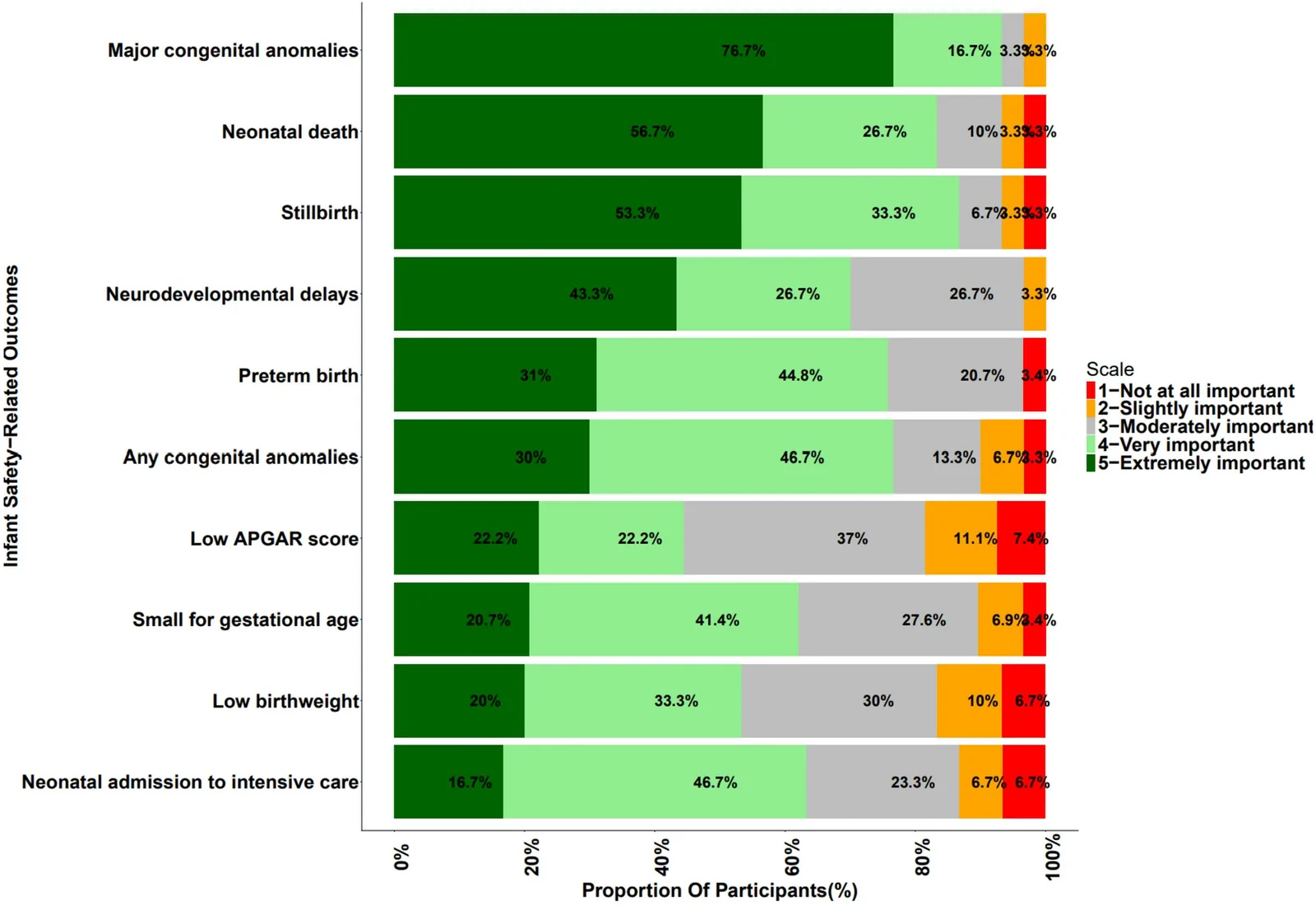
Stakeholder opinions on the relevance of infant outcomes to assess the safety of antimalarials used in the treatment of malaria in the first trimester of pregnancy

The majority of stakeholders identified major congenital anomalies (28/30, 94%) and stillbirth (26/30, 87%) as extremely or very important infant safety outcomes, given the plausible link between antimalarial exposure in the first trimester and these outcomes (Fig. 6). Neonatal death, especially within the first seven days of life, was also considered a key indicator (25/30, 83%), serving as a proxy for early infant death when longer-term follow-up is challenging. Most stakeholders (≥ 50%) rated the remaining infant outcomes as important, except for low Apgar score (13/30, 43%).

Categorisation by participant’s location’s endemicity revealed broad agreement on the importance of most infant outcomes. However, neurodevelopmental delays and low Apgar scores received less support among non-malaria-endemic stakeholders (4/10, 40%, and 1/10, 10%, respectively) compared to malaria-endemic stakeholders (17/20, 85%, and 11/20, 65%, respectively; Figure S9).

There was no significant difference across stakeholder groups, with most stakeholders (≥ 50%) agreeing on the importance of infant outcomes, except for low Apgar score and immediate side effects, which were rated less important by clinical researchers (Figure S10).

**Fig. 7:**
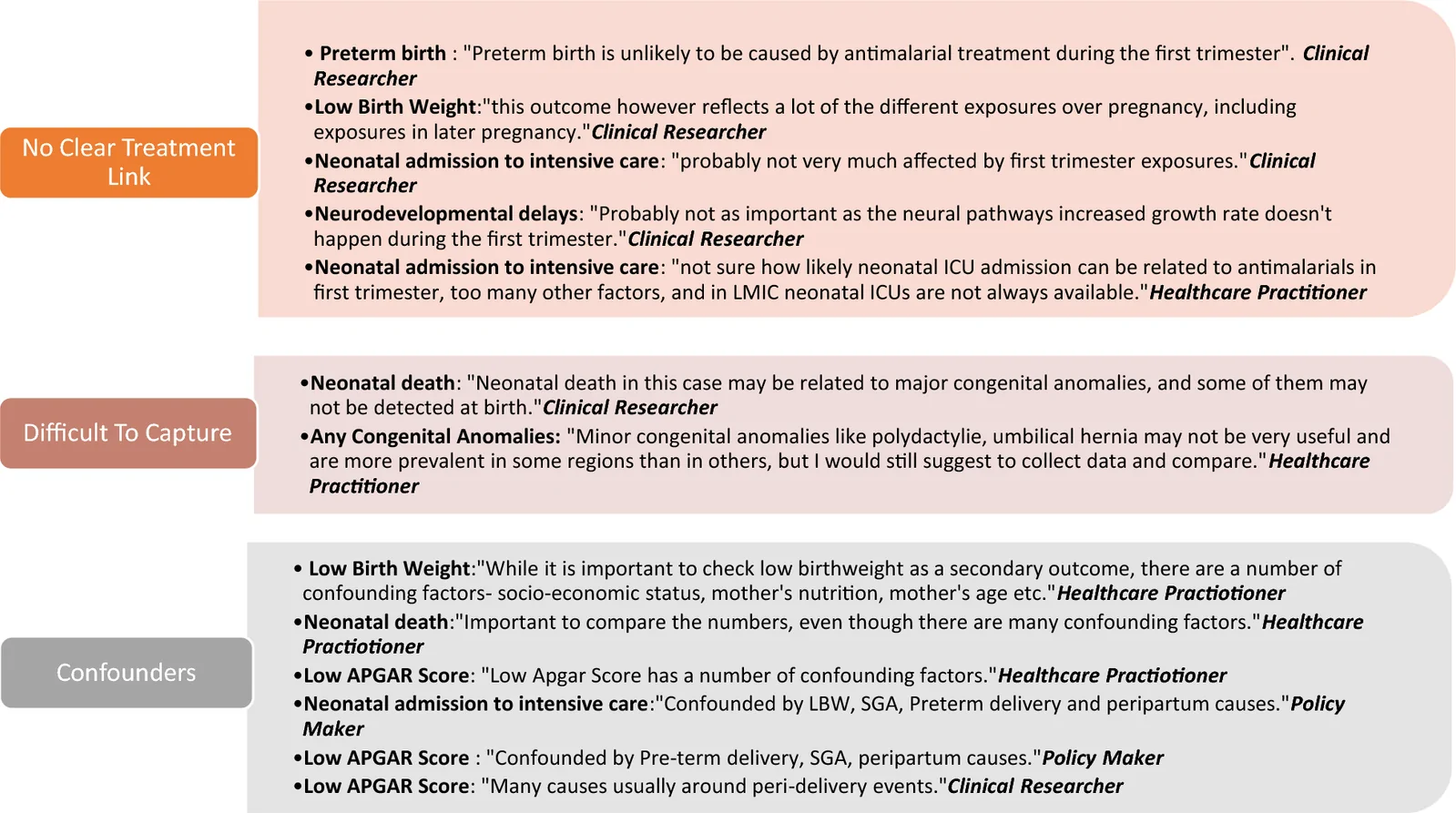
Common themes in stakeholder views on infant-related safety outcomes in the context of first-trimester malaria treatment trials

Figure 7 highlights the reasoning behind stakeholder selection of key infant safety-related outcomes. Many stakeholders noted that outcomes such as low Apgar score, LBW, neonatal intensive care admission, PTB, and neurodevelopmental delays are influenced by multiple risk factors and may not be directly related to antimalarial treatment in the first trimester. Additional challenges were raised regarding the competing risk of certain outcomes. For instance, a clinical researcher suggested that neonatal death may be related to undetected congenital anomalies and is, therefore, important to capture as well.

#### Safety endpoint ranking

**Fig. 8:**
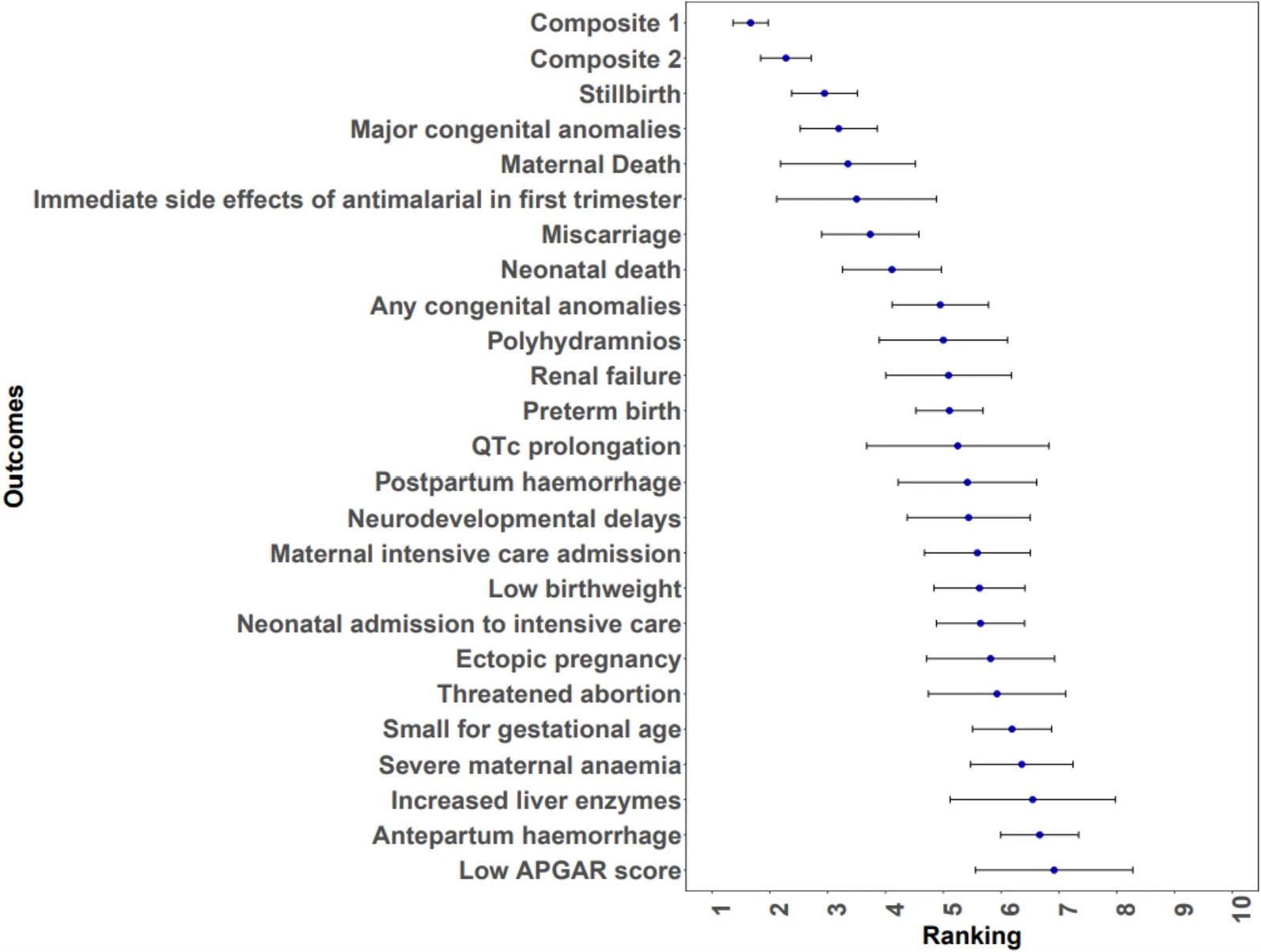
Stakeholder opinions on the importance of safety endpoints for first-trimester malaria treatment trials. Endpoints are ranked from 1 (most important) to 10 (least important); mean scores are shown as blue dots with 95% confidence intervals as whiskers)

**Fig. 9:**
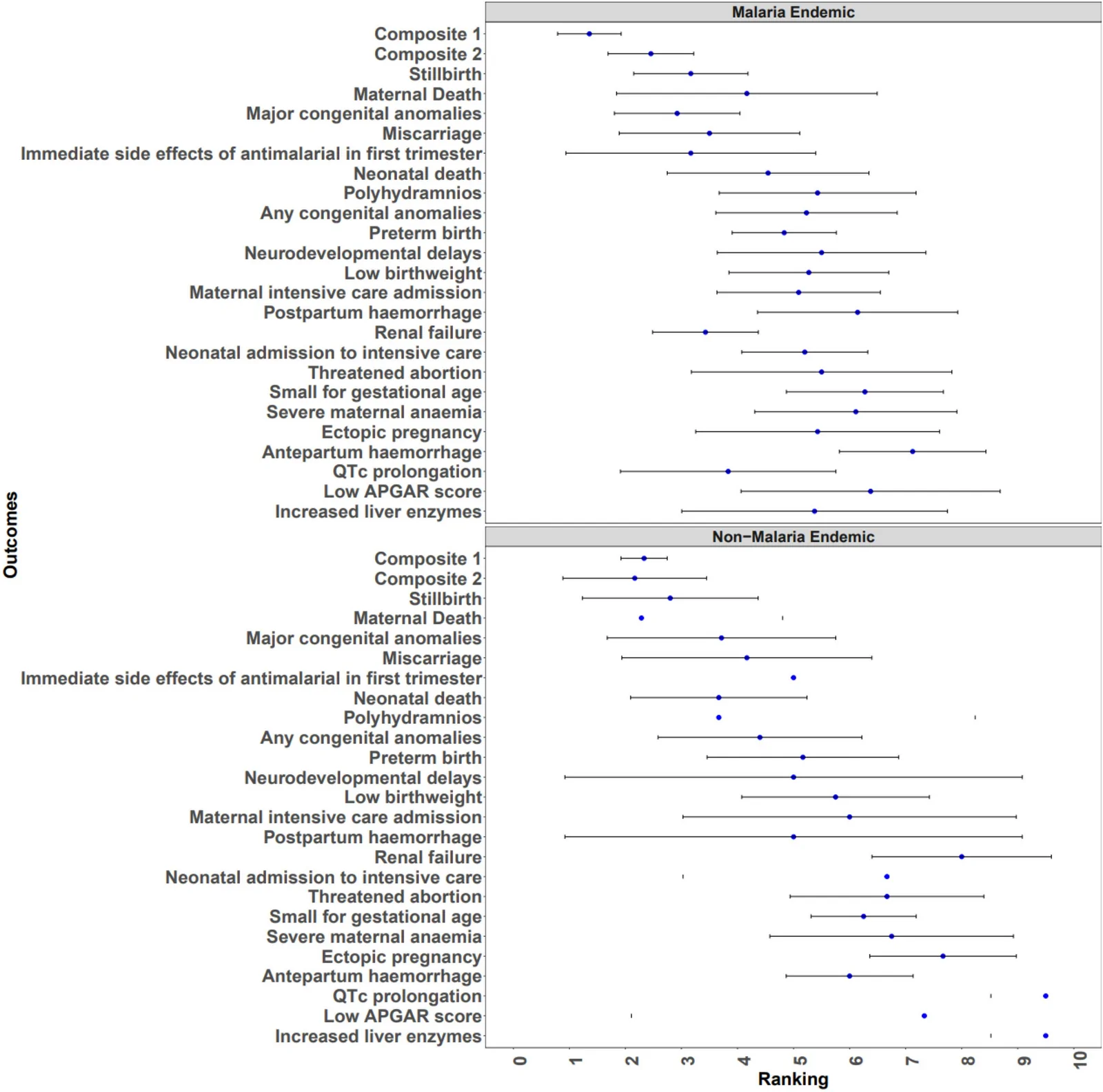
Ranking of safety endpoints stratified by endemicity. Mean scores are shown as blue dots for malaria-endemic and non-malaria-endemic regions, with 95% confidence intervals represented by whiskers. For some outcomes, confidence intervals could not be calculated due to small sample sizes

**Fig. 10:**
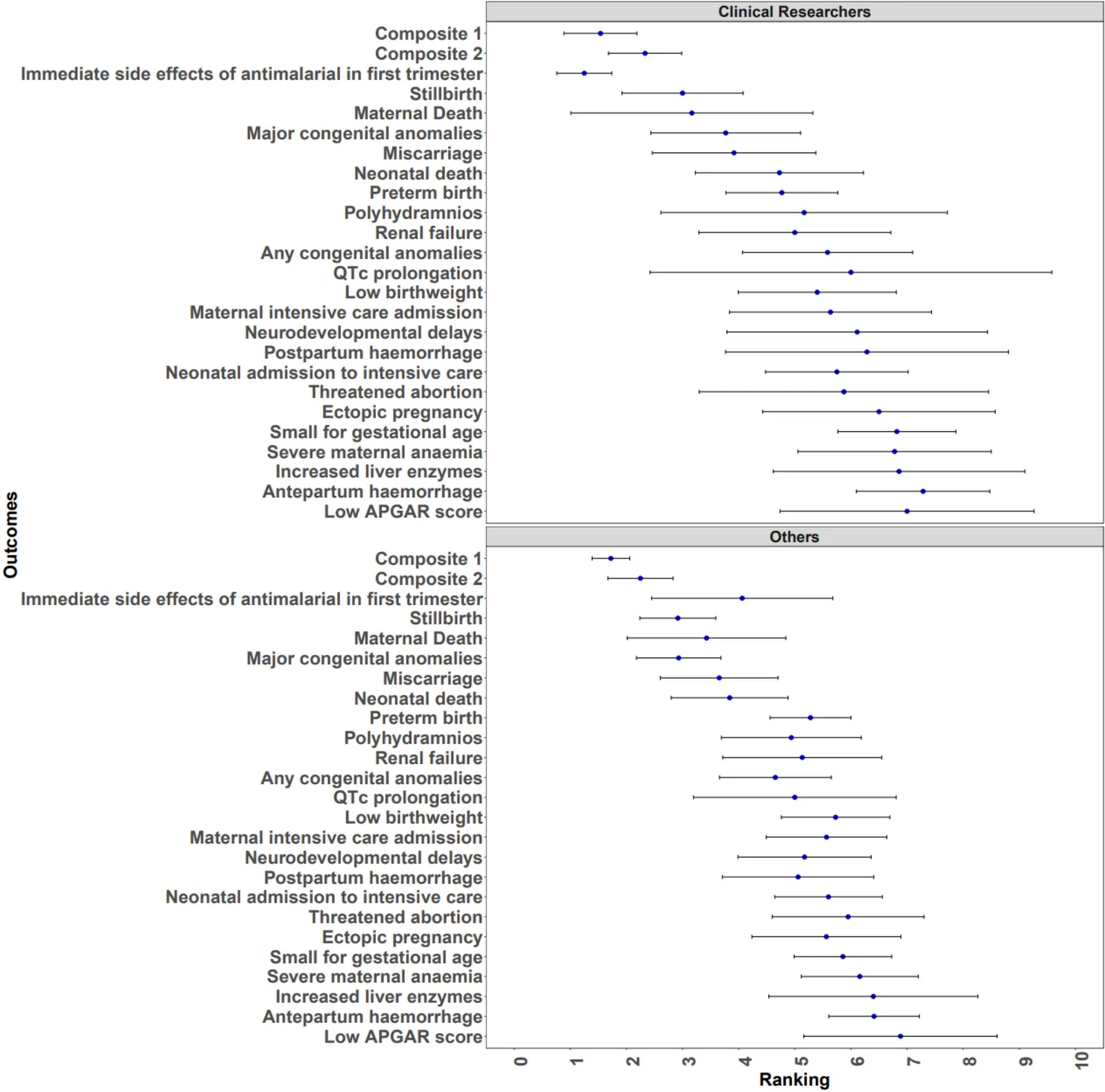
Ranking of safety endpoints stratified by stakeholder group. Mean scores are shown as blue dots for clinical researchers and other stakeholders, with 95% confidence intervals represented by whiskers. For some outcomes, confidence intervals could not be calculated due to small sample sizes

Figure 8 presents the ranking of safety endpoints for first-trimester malaria treatment, with scores ranging from 1 (most important) to 10 (least important); lower scores thus indicate greater perceived importance. Composite 1 (miscarriage, stillbirth, or major congenital anomalies) and Composite 2 (miscarriage, stillbirth, major congenital anomalies, PTB, SGA, LBW, and neonatal death) emerged as the most important safety outcomes, with strong agreement reflected by narrower confidence intervals. The next highest-ranked outcomes were stillbirth, major congenital anomalies, maternal death, immediate side effects of antimalarials in the first trimester, miscarriage, neonatal death, any congenital anomalies, and polyhydramnios. Stratification by malaria endemicity revealed no notable differences in rankings (Fig. 9). However, analysis by stakeholder type (Fig. 10) revealed a slight skew towards higher importance assigned by others compared to clinical researchers for most outcomes, except for ectopic pregnancy, low Apgar score, and polyhydramnios.

#### Safety non-inferiority margins

**Fig. 11:**
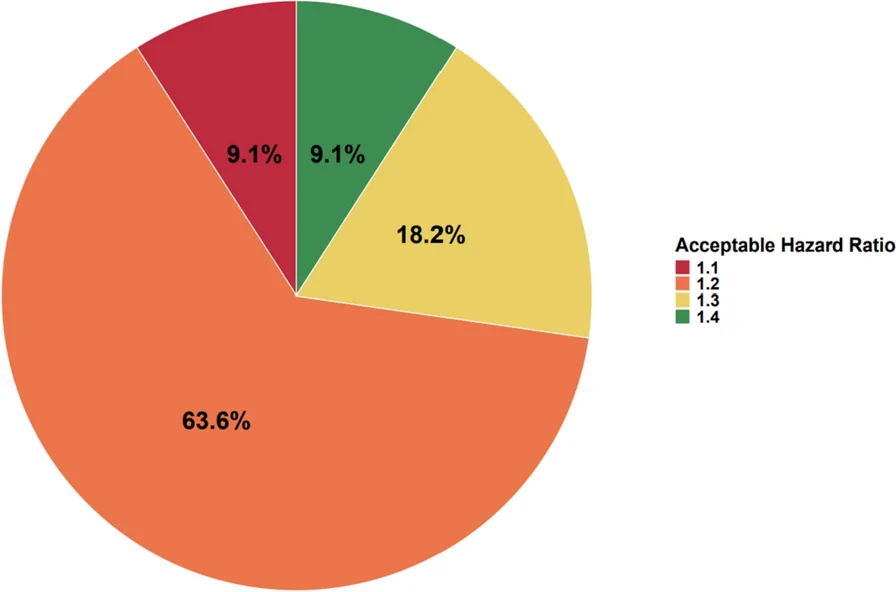
Stakeholder opinions on the acceptable hazard ratio for a new treatment to be considered non-inferior to artemether-lumefantrine for the composite safety endpoint (Composite 1)

Stakeholders were asked to advise on an acceptable non-inferiority margin for the composite endpoint (Composite 1) (see Supplemental File 1 for details on non-inferiority margins for safety endpoints). Figure 11 presents the acceptable hazard ratio (HR) for determining non-inferiority to artemether-lumefantrine in first-trimester pregnancies, assuming equal efficacy and no preclinical safety concerns for the ACT partner drug. Most stakeholders (14/22, 64%) considered an HR of 1.2, equivalent to a 20% increased risk of adverse pregnancy outcomes, to be an acceptable non-inferiority margin. A minority endorsed stricter or more lenient margins: 9% (2/22) suggested an HR of 1.1, and 9% (2/22) endorsed an HR of 1.4. Stakeholders argued that a margin of HR = 1.2 would balance the need to avoid approving treatments that could harm mothers and infants, while also being pragmatically more attainable than smaller non-inferiority margins, which would require prohibitively large sample sizes. Similarly, those who suggested a higher HR (> 1.2) argued that it strikes a balance between achieving a feasible sample size and accepting a modest increased risk of adverse outcomes.

Stakeholders were also asked about their support for including a new antimalarial treatment for first-trimester pregnancies into clinical guidelines if trial results demonstrated an 80% probability that its risk of adverse outcomes was less than 1.4 times that of the standard treatment (Figure S11). Among respondents, 10% (2/21) strongly agreed, 24% (5/21) agreed, 19% (4/21) were neutral, and 48% (10/21) disagreed or strongly disagreed. The high level of disagreement was primarily attributed to concerns about irreversible adverse effects the new treatment may cause, such as miscarriage, congenital anomalies, and stillbirth. However, some stakeholders suggested that a wider margin might be acceptable if the benefit outweighed the risks and the outcomes were less severe. Stakeholders also argued that robust post-approval surveillance of the new treatment would be essential to mitigate risks, although pharmacovigilance systems are often lacking in malaria-endemic countries.

## Discussion

In the absence of standardised measures for assessing the introduction of new antimalarial drugs, methods such as the RT-Delphi survey can provide reliable insights into identifying key safety endpoints and appropriate non-inferiority margins. This study suggests that composite safety endpoints are widely endorsed for assessing antimalarial treatment in first-trimester pregnancy, with stronger support for a focused composite endpoint including miscarriage, stillbirth, and major congenital anomalies than for a broader composite endpoint including infant outcomes such as PTB, SGA, LBW, and neonatal death. The former was deemed most appropriate, as it directly captures the core embryo-fetal risks associated with early pregnancy exposure and potential teratogenicity. The broader composite could serve as a key secondary outcome to capture a wider range of adverse outcomes associated with malaria in pregnancy, although it may compromise specificity by including events less clearly linked to first-trimester drug effects.

Stakeholders emphasised the statistical benefits of composite endpoints, specifically for rare outcomes. However, some expressed concerns regarding the equal weighting of outcomes with varying severity, echoing broader discussions in the obstetric literature about the challenges of interpreting composite perinatal outcomes [21]. Stakeholders also noted the limitations of composite safety endpoints, including the need to consider the individual components separately.

Among individual maternal endpoints, maternal death, miscarriage, immediate side effects, and severe maternal anaemia were selected as crucial safety endpoints as these are clinically severe, potentially caused by first-trimester antimalarial treatment, and feasible to measure. Other outcomes, such as maternal intensive care admission, renal failure, and increased liver enzymes, were considered potentially related to the timing of antimalarial exposure in the first trimester and received varying levels of stakeholder endorsement. The limited support for antepartum and postpartum haemorrhage was attributed to their occurrence later in pregnancy, making them less relevant to first-trimester treatment effects. Ectopic pregnancy, QTc prolongation, and polyhydramnios were considered less relevant as the ascertainment of these outcomes is limited by diagnostic capacity in resource-limited settings, and their biological plausibility for being associated with treatment in the first trimester is weak. Similar challenges have been noted in a previous Delphi study when selecting standardised safety outcomes, as observational research on pregnancy medication safety using primary data may be hindered by under-ascertainment of some pregnancy outcomes [22]. Furthermore, the outcomes prioritised in this survey reflect stakeholder views on what should be measured, but causal attribution of these outcomes to antimalarial treatment, rather than to underlying malaria, depends on well-designed prospective studies with active comparators that address confounding by indication.

For infant outcomes, strong agreement was observed around the importance of major congenital anomalies, stillbirth, and neonatal death, consistent with established causal pathways following exposure in the first trimester [23]. Outcomes such as LBW, SGA, and neonatal intensive care admission were considered important but with less clear causal pathways. Conversely, low Apgar scores and neurodevelopmental delays received mixed support and were generally considered less important.

In terms of ranking, the two composite safety endpoints, stillbirth, major congenital anomalies, and maternal death, emerged as the top five most important outcomes. Polyhydramnios was ranked relatively highly (10th out of 25 outcomes), despite receiving a lower importance rating on the 5-point Likert scale. Although none of the stakeholders explicitly cited this reason, the inclusion of this outcome may reflect evidence from the literature suggesting that polyhydramnios is a clinically meaningful marker of underlying congenital anomalies, such as renal anomalies, while uncertainty remains regarding its direct association with antimalarial treatment.[24]

The majority of stakeholders suggested that a hazard ratio of 1.2 was an acceptable non-inferiority margin for Composite 1. They suggested this margin provides a pragmatic benchmark, balancing the feasibility of trial design with patient safety. Delphi respondents were shown illustrative sample size estimates indicating that a margin of 1.2 would require enrolment of approximately 6,000 first-trimester malaria-positive participants to achieve 80% power for the proposed composite endpoint (Supplemental File 1, Question 3). Despite this substantial sample size, many still viewed 1.2 as an appropriate compromise. However, achieving this sample size would require extensive resources (multi-site and multi-year efforts), making such a trial challenging to implement.

Nearly half (48%) of respondents disagreed with a non-inferiority margin of 1.4, as it implies an unacceptably high potential increase in adverse pregnancy outcomes. In practice, however, trials powered for a non-inferiority margin of 1.4 are more feasible and could provide an initial level of reassurance regarding the safety of alternative antimalarials in early pregnancy. Establishing such evidence is important in the context of multiple first-line therapies (MFTs), a strategy designed to prevent and delay the emergence of antimalarial drug resistance, and already successfully implemented for other infectious diseases such as tuberculosis and HIV [25].

Ruling out a 20% increase in adverse pregnancy outcomes would require meta-analysis of several such trials and/or large-scale post-marketing studies. However, if the field continues to demand such stringent safety margins, progress on generating evidence will stall, ultimately leaving pregnant women and their infants at greater risk due to the persistent lack of reliable data on the relative safety of antimalarial treatments. Policymakers will therefore need to make pragmatic, evidence-informed decisions about which treatments can be recommended in the first trimester, based on accumulating evidence that recognises the need to diversify the available antimalarials, including MFTs, in the general population, and to mitigate risks associated with exposure in early gestation before a pregnancy is recognised.

Previous reviews of non-inferiority trials have highlighted the same challenge: balancing what is ethically acceptable with what statistical power is practically achievable [26]. Given the absence of validated methods for defining safety margins in pregnancy trials, more research is needed to base these limits on real data rather than expert opinion alone. This mirrors broader methodological gaps. A systematic review by Johnson et al*.*[27] underscored the lack of standardised approaches to eliciting expert beliefs; the review authors concluded, however, that structured elicitation methods can reduce bias and improve transparency. Building on this, non-inferiority designs may provide a basis for transparent safety margins in malaria pregnancy trials, especially in the absence of historical data.

### Implications for trial design

Our findings suggest that trial protocols for first-trimester malaria treatment should:

1. Consider a composite endpoint including miscarriage, stillbirth, and major congenital anomalies as the primary safety endpoint, with a broader composite including additional neonatal outcomes (LBW, PTB, SGA, and neonatal death) as a secondary outcome.
2. Include critical individual safety outcomes, specifically maternal death, miscarriage, severe maternal anaemia, major congenital anomalies, stillbirth, and neonatal death when evaluating the safety of antimalarial exposure in the first trimester. Outcomes such as ectopic pregnancy, QTc prolongation, polyhydramnios, LBW, SGA, and neonatal intensive care admission may be considered of lesser importance, partly due to their uncertain relationship with in utero antimalarial exposure.
3. Strengthen global coordination to achieve the target of a non-inferiority margin of HR = 1.2, which will require pooled analyses of multiple trials and/or large-scale surveillance studies.

### Strengths and limitations

This is the first RT-Delphi study to establish the importance of specific safety outcomes in malaria treatment trials during pregnancy, informed by stakeholders from various regions. The survey’s strong representation of clinical researchers with extensive experience ensures that the recommendations are grounded in expert opinion.

Limitations of this study include a modest response rate (56%) (within the acceptable range for Delphi studies) and a relatively small sample size, with underrepresentation of some groups. Future surveys could benefit from greater representation of stakeholders, such as regulators, ethicists, pharmacovigilance and safety experts, and policymakers. The RT-Delphi approach involved reflexive collaboration, as stakeholders could review their answers alongside those of other respondents. This allows respondents to reflect on their answers, but risks leading them to follow the majority rather than their own independent judgment. In practice, however, 40% (12/30) of participants did not return after their initial submission, limiting the extent of iterative reflection across the full set of responses, particularly for earlier participants (Supplemental File 2, Figure S14). Additionally, interpreting stakeholder responses is inherently subjective, relying on the majority to identify areas of agreement and disagreement.

## Conclusions

This study provides valuable insights into selecting safety outcomes and non-inferiority margins for antimalarial treatment trials in first-trimester pregnancy. By engaging stakeholders from diverse backgrounds, we identified critical safety endpoints for assessing antimalarials in the first trimester. Stakeholders favoured a conservative non-inferiority margin (HR = 1.2), underscoring the priority placed on maternal and infant safety; however, such thresholds carry feasibility challenges due to the large sample sizes required. Balancing scientific rigour with practical trial design will be essential to generate the evidence urgently needed to expand antimalarial options in early pregnancy.

## Supplementary Information

Supplementary Material 1.

Supplementary Material 2.

## Data Availability

All data relevant to the study are included in the article or in the online supplemental files.
