# Supplementary material for: Defining safety parameters for first-trimester malaria treatment trials: stakeholder views from Real-Time Delphi survey
Source: Malar J. 2026 Jul 18;25:365. doi: 10.1186/s12936-026-06045-1 (PMC13663266; doi:10.1186/s12936-026-06045-1)
Supplement: Supplementary file 1 [file 12936_2026_6045_MOESM1_ESM.docx]

Defining safety parameters for first-trimester malaria treatment trials: expert views from Real-Time Delphi survey

# **Questionnaire**

# 1. Introduction

Robust evidence on the benefit-harm profile of a range of antimalarials in the first trimester of pregnancy is urgently needed. We propose a novel approach using a multicentre open-label, non-inferiority randomised controlled trial of the safety, tolerability, and efficacy of antimalarials for treating uncomplicated *P. falciparum* malaria in the first trimester of pregnancy using a Bayesian adaptive randomised platform design.

The following questions pertain to the trial design, and your contributions will be instrumental in refining the study.

# 2. Safety Endpoints

a. **Composite safety endpoint**

Composite endpoints that encompass both maternal and fetal/neonatal health outcomes are increasingly used in pregnancy studies,^1,2-6^ accounting for the complex interdependence between the mother and fetus outcomes. However, there is a lack of standardisation in defining these composite outcomes.

Drawing from animal reproductive toxicology studies of the artemisinins, we propose using a composite endpoint that combines pregnancy loss (including miscarriages and stillbirths) and major congenital anomalies as the primary safety outcome. To a degree, these are competing outcomes because severe congenital anomalies that are incompatible with life will result in miscarriage or stillbirth. It has been suggested that up to 85% of miscarriages involve foetal malformations.^7,8^ and that 26-35% of stillbirths are associated with congenital anomalies.^9-11^ Using composite endpoints is one approach to handling competing risks. An unweighted composite is proposed as the clinical severity of these three outcomes is considered comparable.

The use of a composite endpoint has the advantage of using a single outcome that summarises the net effect of treatment, both in terms of efficacy (in terms of protection against malaria-induced pregnancy loss and possibly congenital anomalies linked to high fevers) and safety (potentially drug-induced pregnancy loss or congenital anomalies). It also has the important added benefit of increasing the power of studies because it occurs more frequently than its individual components, allowing for more precision for a given sample size and potentially reducing the required participant numbers or study duration while maintaining the ability to detect clinically meaningful effects.

Each component of the composite endpoint will also be analysed separately as a secondary endpoint to ensure that the effects of one component do not mask the effects of the others.

**Question 1:**

i. To what degree do you agree with the following statement: "Using a composite safety endpoint that includes miscarriage, stillbirth, or major congenital anomalies is informative and appropriate to assess the safety of artemisinin-based combination therapies (ACTs) antimalarials during the first trimester of pregnancy."?

| **1-*Strongly Disagree*** | **2-*Disagree*** | **3-*Neither Agree nor Disagree*** | **4-Agree** | **5-*Strongly Agree*** |
| --- | --- | --- | --- | --- |
|  |  |  |  |  |

ii. Please explain why you chose this answer in the box below.

b. **Question 2: Key safety endpoints**

iii. Please rate the importance of each outcome in the table below for assessing the safety of antimalarials used in the first trimester of pregnancy on a scale from 1 to 5, where 1 is not important, and 5 is extremely important. After rating each outcome, rank the top 10 in order of importance, assigning rank 1 to the most important outcome, rank 2 to the next most important, and so on, across maternal and infant domains. Provide additional comments or rationale for your ratings and rankings in the comment by clicking "Please provide an explanation for your choice ". Suggest any other safety outcome missing from the list in the space provided.

| **Domain** | **Endpoint** | **Definition** | **Importance (1-5)** | **Comment** | **Ranking (top 10)** |
| --- | --- | --- | --- | --- | --- |
| Maternal/Infant | Composite miscarriage, stillbirth or major congenital anomalies | For the definition of each of these components, see below |  |  |  |
| Maternal/Infant | Composite miscarriage, stillbirth, major congenital anomalies, preterm birth, small-for-gestational age, low birthweight or neonatal deaths. | For the definition of each of these components, see below |  |  |  |
| Maternal | Maternal Death | death of the mother anytime during pregnancy and up to 6w eeks post-delivery |  |  |  |
|  | Severe maternal anaemia | haemoglobin <7 g/dl during pregnancy, at delivery, or within 4-8 weeks postpartum |  |  |  |
|  | Spontaneous abortion/miscarriage | confirmed pregnancy that fails to progress, resulting in death and expulsion of the embryo or unviable fetus (<28 weeks gestation as defined for developing countries). |  |  |  |
|  | Threatened abortion | vaginal bleeding and uterine cramping in an otherwise viable pregnancy <28 weeks of gestation |  |  |  |
|  | Ectopic pregnancy | fertilised egg implants itself outside of the womb |  |  |  |
|  | Antepartum haemorrhage | bleeding from or into the genital tract, occurring from 24+0 weeks of pregnancy and prior to the birth of the baby |  |  |  |
|  | Polyhydramnios | pathological increase of amniotic fluid volume in pregnancy |  |  |  |
|  | Postpartum haemorrhage | blood loss of 500 ml or more within 24 hours after birth |  |  |  |
|  | Maternal intensive care admission | Admission in ICU during pregnancy and up to 6-weeks post-delivery |  |  |  |
|  | Increased liver enzymes | Alanine transaminase (ALT) >45 IU/L.  Aspartate transaminase (AST)> 35 IU/L.  Alkaline phosphatase (ALP) >120 IU/L.  Gamma-glutamyltransferase (GGT)> 30 IU/L.  Bilirubin> 17 micromoles/L. |  |  |  |
|  | Renal failure | Serum Creatinine Levels  > 3 mg/dL (265 μmol/L) or  Blood urea nitrogen (BUN) > 60 mg/dL (21.4 mmol/L) or  Urine output < 400 mL/24 hours in adults despite adequate fluid resuscitation |  |  |  |
|  | QTc prolongation | >greater than 480 ms |  |  |  |
| Infant/Neonatal | Stillbirth | a confirmed pregnancy lasting until 28 weeks of pregnancy, that results in the birth of a baby showing no signs of life (including no heart rate or pulsation of the umbilical cord, no respirations, and no definite movement of voluntary muscles) |  |  |  |
|  | Preterm birth | live birth delivered before 37 weeks completed gestation |  |  |  |
|  | Low birthweight | infant with birthweight <2500gr |  |  |  |
|  | Small for gestational age | infant with birth weight for gestational age below the 10th percentile based on sex-specific growth standards for birth weight |  |  |  |
|  | Any congenital anomalies | Any anomaly that is present at birth |  |  |  |
|  | Major congenital anomalies | anomaly that is present at birth with surgical, medical, or cosmetic importance. |  |  |  |
|  | Neonatal death | deaths among live births during the first 28 completed days of life |  |  |  |
|  | Neonatal admission to intensive care | Infant admission to ICU during the first 28 completed days of life |  |  |  |
|  | Neurodevelopmental delays | Significant deviations from expected age-appropriate developmental milestones across domains such as cognitive, language, and motor skills based on standardised assessment tools like the Bayley Scales of Infant and Toddler Development. |  |  |  |
|  | Low AGPAR score | The Apgar score describes the condition of the newborn infant immediately after birth, and can be measured at 1, 5 and 10 minutes. Low Apgar score <7 |  |  |  |
|  | Any others? |  |  |  |  |

# 3. Non-inferiority Margins for Safety Endpoints

A non-inferiority approach is particularly suited for assessing the safety of new ACTs, such as pyronaridine-artesunate or dihydroartemisinin-piperaquine, in treating uncomplicated malaria during the first trimester, as compared to the current standard of care (artemether-lumefantrine). This method acknowledges that a new treatment does not need to be superior in every aspect to be clinically valuable; rather, it should be similarly efficacious and acceptably safe as existing options whilst potentially offering other benefits such as longer-treatment prophylaxis (e.g. 3-5 vs 2 weeks for artemether-lumefantrine) and simpler treatment regimen (once a day vs twice a day). Moreover, it provides a pragmatic framework for determining whether these other ACTs can be considered a viable future alternative, especially in regions where resistance to artemether-lumefantrine may be emerging, or AL is unavailable as first-line treatment in the general population, or in areas where Multiple First-Line Therapy strategies are implemented for resistance management.

When discussing the safety non-inferiority margin (also referred to as the non-inferiority ratio when expressed as a ratio), we refer to the acceptable difference in safety between the two treatments. We want to ensure that any new medication is as safe, or nearly as safe, as the standard treatment. A pre-specified non-inferiority margin is set to determine how close the new medication's safety must be to the standard of care to conclude non-inferiority. Non-inferiority would be concluded when the upper limit of the 95% confidence interval (CI) lies below the pre-specified non-inferiority margin for safety.

For example, in a safety trial with a non-inferiority margin defined as a hazard ratio (HR) of 1.4, if the estimated HR for adverse pregnancy outcomes is 1.20 (i.e. a 20% increase in safety events) with a 95% CI ranging from 0.95 to 1.35 (see green line in Figure 1), non-inferiority in terms of safety would be established because the upper bound of the CI (1.35) is below the non-inferiority margin of 1.4. Conversely, if the HR is 1.55 with a 95% CI of 1.45 to 1.65, inferiority in terms of safety would be concluded (i.e. the experimental medication would be deemed less safe) because the lower bound of the CI (1.45) exceeds the pre-specified non-inferiority margin of 1.4 (red line in Figure 1). If the HR is 1.20 with a 95% CI of 0.80 to 1.60 (see purple line in Figure 1), the trial would be inconclusive as the confidence interval crosses both 1.0 and 1.4, making it unclear if the new treatment is non-inferior. Lastly, in a scenario where the HR is 1.40 with a 95%CI 1.30 to 1.55 (yellow line), the trial would fail to establish non-inferiority and the results lean towards inferiority as the confidence interval crosses 1.4 and is above 1.0.

Figure 1: potential conclusions that can be drawn from a non-inferiority safety trial with a pre-specified non-inferiority margin of 1.4

Note: This forest plot reflects safety data, not efficacy data. In the context of safety studies, where higher event rates indicate worse outcomes, the interpretation of the non-inferiority margin and confidence interval would be different compared to efficacy studies. For safety outcomes, a hazard ratio (HR) above 1 indicates more adverse pregnancy outcomes with the new intervention (i.e., worse, less safe), while HRs below 1 indicate fewer adverse pregnancy outcomes with the new intervention (i.e., better, safer). This is opposite to the interpretation of efficacy data, where higher values typically represent better outcomes.

We would like you to answer two questions related to non-inferiority margins.

**Question 1: What hazard ratio would you consider acceptable as a non-inferiority margin for a newer treatment to be considered non-inferior to artemether-lumefantrine in terms of safety for inclusion in malaria treatment guidelines for first-trimester pregnancies, assuming that the drugs are equally as effective and that no safety signal was detected in preclinical studies for the ACT partner drug.**

i. Please *indicate in the box below the maximum acceptable increase in risk (i.e. non-inferiority margin), expressed as a hazard ratio (HR) based on the considerations mentioned below.*

*Example: If you consider up to a 30% relative increase acceptable, specify a non-inferiority margin of 1.3; up to a 40% increase would correspond to a non-inferiority margin of 1.4, and so on). Please note that the question relates to the ‘true’ hazard ratio (it is not related to confidence intervals), and an HR of 1.0 is not a possible value for a non-inferiority margin.*

ii. Please explain why you chose this answer in the box below.

**Background Scenario:** Consider a scenario where treatment with artemether-lumefantrine (AL) for uncomplicated malaria in the first trimester is associated with a 14% risk of adverse pregnancy outcomes (defined as miscarriage, stillbirths or major congenital anomalies). Assume the new drugs are as effective as AL (PCR-adjusted cure rate by day 42 >=95% for all drugs) and that no safety signal was detected in preclinical studies for the ACT partner drug.

**Factors to consider**

1. ***Benefit-harm analysis:***

*Microscopy or RDT-confirmed malaria infection in early pregnancy is associated with increased risks of adverse pregnancy outcomes, including miscarriage (first-trimester P. falciparum malaria infection has been associated with 1.6-fold higher risk of miscarriage in Thailand^1^) and stillbirth (malaria infection at any time during pregnancy increased the risk of stillbirth about 1.45-fold^2^), preterm birth and fetal growth restriction and severe maternal anaemia.^3^*

2. ***Potential advantages of newer treatments:***

*Newer treatments, like pyronaridine-artesunate and dihydroartemisinin-piperaquine, may offer benefits over artemether-lumefantrine, such as longer post-treatment prophylaxis and once-daily instead of twice-daily dosing.*

3. **Availability, accessibility and patient preference**:

*The availability of alternative options to treat malaria in the first trimester increases accessibility, especially in resource-limited areas, and empowers pregnant patients with more choices in their treatment. This allows for personalised care that considers individual health needs and local drug availability.*

4. ***Implications for study design and sample size***

*Selecting an appropriate non-inferiority margin also requires considering practical constraints, including available funding, study feasibility, duration, and the critical need for timely evidence.*

*The selection of the non-inferiority ratio substantially influences the required sample size: Stricter non-inferiority margins (e.g., 1.1) require considerably larger sample sizes than more lenient margins (e.g., 1.5 or 2) (see Figure 2).*

*Example: In a two-arm non-inferiority trial using a 1:1 allocation with 80% power and a 14.0% risk in the control arm and allowing for 15% drop-out, a non-inferiority ratio of 1.1 requires 29,044 malaria-infected participants in their first trimester of pregnancy, whereas a non-inferiority ratio of 1.5 requires 1,606 and 2.0 requires only 550 women.*

Figure 2. Sample size requirements for a 2-arm trial (1:1 allocation) by non-inferiority margin, expressed as hazard ratio (HR), assuming one-sided alpha (0.025), 80% power, and rate of 14% in control.

**Question 2:** **Based on the information below, would you support adding a new malaria treatment for first-trimester pregnancies to the guidelines if a clinical trial shows there is an 80% probability its risk of adverse pregnancy outcomes is less than 1.4 times that of the current standard treatment, artemether-lumefantrine,** **assuming that the drugs are equally as effective and that no safety signal was detected in preclinical studies for the ACT partner drug?**

| **1-*Strongly Disagree*** | **2-*Disagree*** | **3-*Neither Agree nor Disagree*** | **4-Agree** | **5-*Strongly Agree*** |
| --- | --- | --- | --- | --- |
|  |  |  |  |  |

i. Please explain why you chose this answer in the box below.

**Background to Scenario:**

Based on Bayesian simulations, our estimates suggest that the current funding is sufficient for a trial with a sample size large enough to achieve the following: the study would have at least an 80% chance (i.e. posterior probability) of detecting a true hazard ratio for the primary safety endpoint (encompassing miscarriage, stillbirth, or major congenital anomalies) higher than 1.4 when compared to artemether-lumefantrine. This is equivalent to 80% power in frequentist statistical terms.

This implies that if the new treatment increases the risk by more than 1.4 fold (which would mean more than about 20 out of every 100 women experiencing these outcomes compared to 14 with artemether-lumefantrine), we have an 80% chance of detecting this increase. This 80% chance is what statisticians call a 'posterior probability'. After we have conducted the study and analysed the results, it means we will have an 80% level of confidence in our conclusion about whether the new treatment's risk is higher than 1.4 times that of artemether-lumefantrine or not.

If the hazard ratio is 1.4 times or less, it does not necessarily mean the new treatment is exactly as safe as the current one. Rather, it means we can be reasonably confident that any increase in risk, if it exists, is likely less than this 1.4 threshold.

Again, assume the new drug is as effective as artemether-lumefantrine (PCR-adjusted cure rate by day 42 >=95% for all drugs) and that there was no safety signal detected in preclinical studies for the ACT partner drug.

**Factors to Consider in addition to those outlined in question 1 above**

1. **Clinical Significance vs. Statistical Significance:**

- Consider what magnitude of potential increased risk you deem clinically meaningful, even if not statistically significant (see also question 1 above). Specifically, is a potential 1.4 fold increase in adverse pregnancy outcomes an acceptable risk, given the current challenges in treating malaria during pregnancy?
- Consider the implications of potentially underestimating the risk (Type II error: where the treatment may be mistakenly deemed as safe or non-inferior when, in fact, it poses a greater risk than the comparator) in the context of widespread use of the new treatment. This could occur if the non-inferiority margin is set too high or if a wide confidence interval (CI) is accepted (with frequentist approaches), leading to greater uncertainty in the results.
- Certainty of evidence: How does the 80% posterior probability influence your confidence in making a recommendation?

2. **Practical Considerations:**

- While detecting lower differences in risk provides more reassurance, they require larger studies, which have implications for:
- Study duration
- Cost
- Feasibility in the target population
- Timeliness of evidence generation

3. **Existing Safety Data:**

- Consider how this uncertainty would be interpreted in the context of existing safety data from 2^nd^/3^rd^-trimester populations, pre-clinical studies or pregnancy exposure registries of inadvertent first-trimester exposures.

# References

1.	Hernan MA, Schisterman EF, Hernandez-Diaz S, 2014. Invited commentary: composite outcomes as an attempt to escape from selection bias and related paradoxes. Am J Epidemiol 179: 368-70.

2.	2018. HYPATIA: A prospective randomised controlled trial of HYdroxychloroquine to improve Pregnancy outcome in women with AnTIphospholipid Antibodies. Available at: <https://www.clinicaltrialsregister.eu/ctr-search/trial/2016-002256-25/GB>. Accessed 09/09/20.

3.	Thom EA, Rice MM, Saade GR, Reddy UM, Eunice Kennedy Shriver National Institute of Child H, Human Development Maternal-Fetal Medicine Units N, 2016. What we have learned about the design of randomised trials in pregnancy. Semin Perinatol 40: 328-34.

4.	Panariello N, Jurczak A, Spector J, Kumar V, Semrau K, 2019. Coherence in measurement and programming in maternal and newborn health: experience from the BetterBirth trial. J Clin Epidemiol 113: 83-85.

5.	Rubinstein F, Micone P, Bonotti A, Wainer V, Schwarcz A, Augustovski F, Pichon Riviere A, Karolinski A, Antigripal EVASRGEEyV, 2013. Influenza A/H1N1 MF59 adjuvanted vaccine in pregnant women and adverse perinatal outcomes: multicentre study. BMJ 346: f393.

6.	FDA, 2015. Product approval-prescribing information [Package insert]. Gardasil [human papillomavirus quadrivalent (types 6, 11, 16, and 18) vaccine, recombinant], Merck & Co, Inc. . US Department of Health and Human Services FaDA, ed.

7.	Larsen EC, Christiansen OB, Kolte AM, Macklon N, 2013. New insights into mechanisms behind miscarriage. BMC Med 11: 154.

8.	Philipp T, Philipp K, Reiner A, Beer F, Kalousek DK, 2003. Embryoscopic and cytogenetic analysis of 233 missed abortions: factors involved in the pathogenesis of developmental defects of early failed pregnancies. Hum Reprod 18: 1724-32.

9.	Holmes LB, Nasri H, Beroukhim R, Hunt AT, Roberts DJ, Toufaily MH, Westgate MN, 2018. Stillborn Infants: Associated Malformations. Birth Defects Res 110: 114-121.

10.	Horn LC, Langner A, Stiehl P, Wittekind C, Faber R, 2004. Identification of the causes of intrauterine death during 310 consecutive autopsies. Eur J Obstet Gynecol Reprod Biol 113: 134-8.

11.	Mueller RF, Sybert VP, Johnson J, Brown ZA, Chen WJ, 1983. Evaluation of a protocol for post-mortem examination of stillbirths. N Engl J Med 309: 586-90.

12.	Gamble C, Ekwaru JP, ter Kuile FO, 2006. Insecticide-treated nets for preventing malaria in pregnancy. Cochrane Database Syst Rev: CD003755.

13.	Moore KA, Simpson JA, Scoullar MJL, McGready R, Fowkes FJI, 2017. Quantification of the association between malaria in pregnancy and stillbirth: a systematic review and meta-analysis. Lancet Glob Health 5: e1101-e1112.

14.	Grant RL, 2014. Converting an odds ratio to a range of plausible relative risks for better communication of research findings. BMJ 348: f7450.
