# Supplementary material for: Defining safety parameters for first-trimester malaria treatment trials: stakeholder views from Real-Time Delphi survey
Source: Malar J. 2026 Jul 18;25:365. doi: 10.1186/s12936-026-06045-1 (PMC13663266; doi:10.1186/s12936-026-06045-1)
Supplement: Supplementary file 2 [file 12936_2026_6045_MOESM2_ESM.docx]

Defining safety parameters for first-trimester malaria treatment trials: expert views from Real-Time Delphi survey- Supplementary file

# Table of Content

[**Figure S1.** Flow of participants in the Delphi survey	3](#_Toc233208850)

[**Figure S2.** Geographic distribution of stakeholders.	4](#_Toc233208851)

[**Figure S3.** Proportion of experts selecting each category (not at all important to extremely important) for Composite 1 (miscarriage, stillbirth, and major congenital anomalies) used for the evaluation of antimalarial safety in the first trimester, stratified by endemicity.	5](#_Toc233208852)

[**Figure S4.** Proportion of experts selecting each category (not at all important to extremely important) for Composite 1 (miscarriage, stillbirth, and major congenital anomalies) used for the evaluation of antimalarial safety in the first trimester, stratified by stakeholder group.	6](#_Toc233208853)

[**Figure S5**. Proportion of experts selecting each category (not at all important to extremely important) for Composite 2 (miscarriage, stillbirth, major congenital anomalies, preterm birth, small-for-gestational-age, low birth weight, and neonatal death) used for the evaluation of antimalarial safety in the first trimester, stratified by malaria endemicity.	7](#_Toc233208854)

[**Figure S6.** Proportion of experts selecting each category (not at all important to extremely important) for Composite 2 (miscarriage, stillbirth, major congenital anomalies, preterm birth, small-for-gestational-age, low birth weight, and neonatal death) used for the evaluation of antimalarial safety in the first trimester, stratified by stakeholder group.	8](#_Toc233208855)

[**Figure S7.** Experts' views on the relevance of maternal outcomes to assess the safety of antimalarials used in the treatment of malaria in the first trimester of pregnancy, stratified by endemicity.	9](#_Toc233208856)

[**Figure S8.** Experts' views on the relevance of maternal outcomes to assess the safety of antimalarials used in the treatment of malaria in the first trimester of pregnancy, stratified by stakeholder group.	10](#_Toc233208857)

[**Figure S9.** Experts' views on the relevance of infant outcomes to assess the safety of antimalarials used in the treatment of malaria in the first trimester of pregnancy, stratified by endemicity.	11](#_Toc233208858)

[**Figure S10.** Experts' views on the relevance of infant outcomes to assess the safety of antimalarials used in the treatment of malaria in the first trimester of pregnancy, stratified by stakeholder group.	12](#_Toc233208859)

[**Figure S11**. Stakeholder support for adding a new malaria treatment for first-trimester pregnancies to clinical guidelines under a probabilistic evidence scenario.	13](#_Toc233208860)

[**Figure S12**. Stakeholder engagement timeline in the Real-Time Delphi survey.	14](#_Toc233208861)

**Table S1**. Checklist of the CREDES guidelines for conducting and reporting Delphi studies…………………………………………………………………………………………………………..15

# Supplemental figures

**Figure S1.** Flow of participants in the Delphi survey

**Figure S2.** Geographic distribution of stakeholders.

|  |  |
| --- | --- |
|  | |
|  | |

**Figure S3.** Proportion of experts selecting each category (not at all important to extremely important) for Composite 1 (miscarriage, stillbirth, and major congenital anomalies) used for the evaluation of antimalarial safety in the first trimester, stratified by endemicity.

**Figure S4.** Proportion of experts selecting each category (not at all important to extremely important) for Composite 1 (miscarriage, stillbirth, and major congenital anomalies) used for the evaluation of antimalarial safety in the first trimester, stratified by stakeholder group.

**Figure S5**. Proportion of experts selecting each category (not at all important to extremely important) for Composite 2 (miscarriage, stillbirth, major congenital anomalies, preterm birth, small-for-gestational-age, low birth weight, and neonatal death) used for the evaluation of antimalarial safety in the first trimester, stratified by malaria endemicity.

**Figure S6.** Proportion of experts selecting each category (not at all important to extremely important) for Composite 2 (miscarriage, stillbirth, major congenital anomalies, preterm birth, small-for-gestational-age, low birth weight, and neonatal death) used for the evaluation of antimalarial safety in the first trimester, stratified by stakeholder group.

**Figure S7.** Experts' views on the relevance of maternal outcomes to assess the safety of antimalarials used in the treatment of malaria in the first trimester of pregnancy, stratified by endemicity.

**Figure S8.** Experts' views on the relevance of maternal outcomes to assess the safety of antimalarials used in the treatment of malaria in the first trimester of pregnancy, stratified by stakeholder group.

**Figure S9.** Experts' views on the relevance of infant outcomes to assess the safety of antimalarials used in the treatment of malaria in the first trimester of pregnancy, stratified by endemicity.

**Figure S10.** Experts' views on the relevance of infant outcomes to assess the safety of antimalarials used in the treatment of malaria in the first trimester of pregnancy, stratified by stakeholder group.

**Figure S11**. Stakeholder support for adding a new malaria treatment for first-trimester pregnancies to clinical guidelines under a probabilistic evidence scenario.

Stakeholders were asked to indicate their level of support on a 5-point Likert scale (1 = strongly oppose, 5 = strongly support) for adding a new treatment if a clinical trial demonstrated an 80% probability that its risk of adverse pregnancy outcomes was less than 1.4 times that of the current standard treatment, artemether-lumefantrine. Responses are presented as percentages across stakeholder groups.

**Figure S12**. Stakeholder engagement timeline in the Real-Time Delphi survey.

Horizontal bars represent each participant's engagement period from first access (●) to last access (○). Bar colour indicates visit frequency: red (single visit), yellow (2–3 visits), green (4–5 visits), and blue (6+ visits). Weekly periods are indicated along the horizontal axis. Early entrants (Weeks 1–2) demonstrated longer engagement spans and higher revisitation rates compared to later participants who had access to accumulated panel input.

# Supplemental tables

**Table S1.** Checklist of the CREDES guidelines for conducting and reporting Delphi studies

| **Section / Topic** | **Item #** | **Recommendation** | **Reported on page(s) #** |
| --- | --- | --- | --- |
| Purpose and rationale | 1 | The purpose of the study should be clearly defined and demonstrate the appropriateness of the use of the Delphi technique as a method to achieve the research aim. A rationale for the choice of the Delphi technique as the most suitable method needs to be provided | 3 |
| Expert panel | 2 | Criteria for the selection of experts and transparent information on recruitment of the expert panel, socio-demographic details, including information on expertise regarding the topic in question, (non)response and response rates over the ongoing iterations should be reported | 4 |
| Description of the  methods | 3 | The methods employed need to be comprehensible; this includes information on preparatory steps, piloting of material and survey instruments, design of the survey instrument(s), the number and design of survey rounds, methods of data analysis, processing and synthesis of experts’ responses to inform the subsequent survey round and methodological decisions taken by the research team throughout the process | 4 |
| Procedure | 4 | Flow chart to illustrate the stages of the Delphi process, including a preparatory phase, the actual ‘Delphi rounds’, interim steps of data processing and analysis, and concluding steps | Suppl. |
| Definition and  attainment of  consensus | 5 | It needs to be comprehensible to the reader how consensus was achieved throughout the process, including strategies to deal with non-consensus | 4 and 5 |
| Results | 6 | Reporting of results for each round separately is highly advisable in order to make the evolving of consensus over the rounds transparent.  This includes figures showing the average group response, changes between rounds, as well as any modifications of the survey instrument such as deletion, addition or modification of survey items based on previous rounds | NA |
| Discussion of  limitations | 7 | Reporting should include a critical reflection of potential limitations and their impact of the resulting guidance | 11 |
| Adequacy of  conclusions | 8 | The conclusions should adequately reflect the outcomes of the Delphi study with a view to the scope and applicability of the resulting practice guidance | 11 |
| Publication and  dissemination | 9 | The resulting guidance should be clearly identifiable from the publication, including recommendations for transfer into practice and implementation. If the publication does not allow for a detailed presentation of either the resulting practice guidance or the methodological features of the applied Delphi technique, or both, reference to a more detailed presentation elsewhere should be made (e.g. availability of the full guideline from the authors or online; publication of a separate paper reporting on methodological details and particularities of the process (e.g. persistent disagreement and controversy on certain issues)). A dissemination plan should include endorsement of the guidance by professional associations and health care authorities to facilitate implementation | NA |

# Supplemental references

1. Jünger S, Payne SA, Brine J, Radbruch L, Brearley SG. Guidance on Conducting and REporting DElphi Studies (CREDES) in palliative care:

Recommendations based on a methodological systematic review. Palliat Med. 2017;31:684–706.
